# Risk of cancer in patients with fecal incontinence

**DOI:** 10.1002/cam4.2509

**Published:** 2019-08-29

**Authors:** Kasper Adelborg, Katalin Veres, Jens Sundbøll, Hans Gregersen, Henrik Toft Sørensen

**Affiliations:** ^1^ Department of Clinical Epidemiology Aarhus University Hospital Aarhus N Denmark; ^2^ GIOME, Department of Surgery The Chinese University of Hong Kong Shatin Hong Kong China

**Keywords:** cancer, epidemiology, fecal incontinence

## Abstract

**Background:**

Fecal incontinence may be an early symptom of cancer, but its association with cancer remains unclear. We examined the risk of selected cancers, including colorectal cancer, other gastrointestinal cancers, hormone‐related cancers, and lymphoma, in patients with fecal incontinence.

**Methods:**

Using Danish population‐based registries, all patients with hospital‐based diagnoses of fecal incontinence during 1995‐2013 were identified. We calculated cumulative incidences of cancers. As a measure of relative risks, we computed standardized incidence ratios (SIRs), that is, the observed number of cancers relative to the expected number based on national incidence rates by sex, age, and calendar year.

**Results:**

Among 16 556 patients with fecal incontinence, the cumulative incidence of colorectal cancers, other gastrointestinal cancers, hormone‐related cancers, and lymphoma each was less than 0.4% after 1 year. It increased to under 3% after 10 years. The SIR for any cancer during 19 years of follow‐up was 1.12 [95% confidence interval (CI), 1.06‐1.19]. The SIRs during the first year were 2.31 (95% CI, 1.65‐3.13) for colorectal cancer, 1.56 (95% CI, 0.89‐2.54) for other gastrointestinal cancers, 1.00 (95% CI, 0.72‐1.35) for hormone‐related cancers, and 2.02 (95% CI, 1.01‐3.61) for lymphoma. Beyond 1 year, the SIR reached unity for other gastrointestinal cancers, hormone‐related cancers, and lymphoma, while a reduced risk was observed for colorectal cancer (SIR = 0.77, 95% CI, 0.59‐0.98).

**Conclusions:**

Fecal incontinence was a marker of cancer, especially gastrointestinal cancers and lymphoma within 1 year, which presumably is driven partly by reverse causation. However, the absolute risks were low. Heightened diagnostic efforts may explain in part the increased short‐term risk of colorectal cancers.

## Graphical abstract

Fecal incontinence was a marker of gastrointestinal cancers and lymphoma within 1 year. However, the absolute cancer risks were low. Our data provided insight into the clinical course following fecal incontinence and may inform screening guidelines.

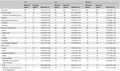

## 1 INTRODUCTION

Fecal incontinence is defined as involuntary loss of rectal contents.^1^ It affects up to 8% of the general population,^2^, ^3^ approaching 50% in study populations of elderly persons and in persons with comorbidities.^4^ It impacts quality of life, has serious consequences for social activities, and poses significant costs related to diagnostic work‐up and treatment.^1^, ^5^ The etiology of fecal incontinence is multifactorial and includes weakness of the anal sphincter, inflammatory bowel disease, irritable colon, childbirth, obesity, diabetes, smoking, anorectal surgery, and neurologic diseases such as Parkinson's disease.^1^, ^5^

Changes in bowel habits such as diarrhea, bloody stools, and constipation are important markers of an active yet undiagnosed cancer.^6^ However, to the best of our knowledge, previous studies have not investigated whether fecal incontinence represents an early symptom of occult cancer. Accumulating evidence suggests that disturbances in gut microbiota, evident in patients with conditions such as fecal incontinence,^7^ may promote carcinogenesis in the gastrointestinal tract and other locations.^7^ Microbiota affect the metabolism of hormones, including estrogens and testosterone,^7^ potentially modifying the risk of hormone‐related cancers. Fecal incontinence also may be linked to cancer through shared risk factors such as smoking and obesity.^8^

To contribute to the understanding of fecal incontinence as a potential risk marker for cancer, we conducted a nationwide population‐based cohort study of patients with fecal incontinence in Denmark and examined their risk of cancers, including colorectal cancers, other gastrointestinal cancers, hormone‐related cancers, lymphoma, and other cancers. We also compared cancer risk in this patient population with that of the general population. Such data may provide insight into the clinical course of patients with fecal incontinence and thereby guide the diagnostic work‐up of these patients.

## 2 METHODS

### 2.1 Setting and design

This study was based on data obtained from Danish administrative and healthcare registries.^9^ The Danish health care system is tax‐funded, ensuring health care for all Danish residents, with free access to hospital treatment and health care provided by general practitioners.^10^ A unique personal identifier is assigned to all Danish residents at birth and to residents upon immigration, which allows for linkage of Danish databases at the individual level.^9^

### 2.2 Fecal incontinence cohort

We searched the Danish National Patient Registry (DNPR) covering all Danish hospitals to identify all patients with a first‐time hospital‐based diagnosis of fecal incontinence between January 1, 1995 and November 30, 2013.^11^ Patients with a prior history of cancer, except nonmelanoma skin cancer, were excluded.^11^ We included both primary and secondary diagnoses, based on both hospital inpatient and outpatient records. The DNPR contains data on all diagnoses and procedures for patients discharged from Danish hospitals, coded according to the *International Classification of Diseases*, *Eighth Revision* (ICD‐8) from 1977 until 1993 and the *Tenth Revision* (ICD‐10) thereafter. Data on hospital outpatient visits and emergency room contacts were added to the database starting in 1995. Reporting to the DNPR is mandatory for both inpatient and outpatient contacts, each recorded with one primary discharge diagnosis and one or more optional secondary diagnoses. Primary diagnoses are the main reason for hospitalization, while secondary diagnoses represent optional diagnoses supplementing the primary diagnosis by, describing the underlying chronic disease that is related to the current patient contact.

### 2.3 Cancer outcomes

To identify incident cancer outcomes, the cohort of patients diagnosed with fecal incontinence was linked to the Danish Cancer Registry (DCR).^12^, ^13^ The following cancer outcomes were selected: (a) any cancer; (b) colorectal cancers including colon, rectosigmoid, and rectal cancers; (c) other gastrointestinal cancers, that is, cancers occurring in the esophagus, stomach, small intestine, anus, liver, gall bladder and biliary tract, and pancreas; (d) hormone‐related cancers, including cancers of the breast, corpus uteri, ovary, and prostate; (e) lymphomas, encompassing Hodgkin malignant lymphoma and non‐Hodgkin malignant lymphoma; and (f) other cancers arising in the oral cavity, larynx, lung, malignant melanoma, kidneys, urinary bladder, membrane of the brain and spinal meninges, and brain, as well as metastases, and nonspecified cancer in lymph nodes.

The DCR has recorded information on all cases of incident cancer since 1943, classified according to ICD‐10.^12^ Completeness of cases finding and data in the DCR has been consistently high, although mandatory reporting to the DCR was first implemented in 1987.

### 2.4 Covariates

Study covariates included age at diagnosis of fecal incontinence (0‐17, 18‐34, 35‐49, 50‐64, and ≥65 years), sex, type of hospital contact (inpatient and outpatient), calendar period of fecal incontinence diagnosis (1995‐2001, 2002‐2009, and 2010‐2013), and presence of comorbid diseases included in the Charlson Comorbidity Index (excluding cancer conditions from the Index), as recorded in the DNPR, from 1977 until the date of a fecal incontinence diagnosis.^14^ We classified comorbidity into three levels: none (0), moderate (1‐2), and severe (3+). Data also were retrieved from the DNPR on diagnostic procedures, including colonoscopy and sigmoidoscopy, anal/perianal surgery and rupture of the perineum requiring surgery, and episiotomy within 3 months before and 4 weeks after the diagnosis of fecal incontinence. In addition data on inflammatory bowel disease, diabetes mellitus, Parkinson's disease, obesity, and child birth within 5 years before the diagnosis of fecal incontinence were obtained from the DNPR. All diagnostic codes used in the study are provided in Tables S1‐S4.

### 2.5 Statistical analyses

We followed patients from the hospital contact date for fecal incontinence until the date of a cancer diagnosis, emigration, death, or November 30, 2013, whichever came first. We computed median follow‐up time with an interquartile range. We characterized the fecal incontinence patients according to age, sex, type of hospital contact, calendar period, Charlson Comorbidity Index score, and performance of a diagnostic procedure. The cumulative incidence of cancer was calculated after 1, 5, and 10 years of follow‐up, accounting for death as competing risk.^15^ As a measure of relative risk, we calculated standardized incidence ratios (SIRs), that is*,* the ratio of observed cancer incidence divided by expected cancer incidence, based on national cancer incidence rates by sex, age (1‐year groups), and calendar year (1‐year groups). Confidence intervals (CIs) were calculated using Byar's approximation, assuming that the observed number of cancers followed a Poisson distribution. Exact 95% CIs were derived when the observed number of cancers was fewer than 10.^16^

To explore the impact of heightened diagnostic efforts, and because fecal incontinence can be a direct consequence of diagnostic or surgical procedures, for example, lower endoscopy (colonoscopy and sigmoidoscopy) or anal/perianal surgery within 3 months before and 4 weeks after the fecal incontinence diagnosis, we stratified the colorectal cancer SIR analyses by these procedures. In this analysis, we followed patients from 4 weeks after the fecal incontinence diagnosis to avoid immortal time bias. To identify potential interactions, subgroup analyses were done by age, sex, type of hospital contact (inpatient vs outpatient and primary vs secondary diagnoses), calendar period, and Charlson Comorbidity Index score. All statistical analyses were performed using SAS version 9.4 (Cary, NC). The study was approved by the Danish Data Protection Agency (record number 1‐16‐02‐1‐08). Studies based on registry data do not require informed consent in Denmark.

## 3 RESULTS

We identified 16 556 patients with a first‐time hospital‐based diagnosis of fecal incontinence. This cohort included 11 220 (68%) women and 5536 (32%) men with a median age of 51 years (interquartile range, 24‐69 years) (Table 1). During total follow‐up time of 87 264 person‐years (median follow‐up time 5 years, interquartile range: 2‐9 years), 1118 patients received a cancer diagnosis. Most patients were diagnosed in the hospital outpatient setting (87%) and had no comorbidities (72%).

**Table 1:** Characteristics of patients with a first‐time diagnosis of fecal incontinence in Denmark, 1995‐2013. Data are numbers (%)

	No. (%)
Total	16 556
Median follow‐up time, y (interquartile range)	5 (2‐9)
Median age, y (interquartile range)	51 (24‐69)
Age at diagnosis of fecal incontinence (y)	
0‐17	3963 (24)
18‐34	1830 (11)
35‐49	2256 (14)
50‐64	3280 (20)
65+	5227 (32)
Sex	
Female	11 220 (68)
Male	5336 (32)
Type of hospital contact	
Inpatient	2167 (13)
Outpatient	14 389 (87)
Calendar period	
1995‐2001	3479 (21)
2002‐2009	7402 (45)
2010‐2013	5675 (34)
Charlson Comorbidity Index score	
None (0)	11860 (72)
Moderate (1‐2)	3772 (23)
High (≥3)	924 (6)
Diagnostic/surgical procedure	
Colonoscopy	1258 (8)
Sigmoidoscopy	2195 (13)
Anal/perianal surgery	705 (4)
Rupture of perineum requiring surgery	91 (0.5)
Episiotomy	18 (0.1)
Other comorbidities	
Inflammatory bowel disease	206 (1)
Diabetes mellitus	818 (4.9)
Parkinson's disease	70 (0.4)
Obesity	710 (4)
Child birth	1895 (11)

Around 20% of patients had an endoscopy during the 3 months before or 4 weeks after their fecal incontinence diagnosis. The 1‐year cumulative incidence of any cancer diagnosis was 1.4% (95% CI, 1.2%‐1.6%). The 5‐year cumulative incidence was 5.9% (5.5%‐6.3%), and the 10‐year cumulative incidence was 10.8% (95% CI, 10.2%‐11.5%). These incidences were slightly higher among women than among men and increased with advancing age. The corresponding SIR for any cancer during the 19 years of follow‐up was 1.12 (95% CI, 1.06‐1.19), while the SIR was 1.32 (95% CI, 1.16‐1.51) within 1 year. After 1 year, a slightly increased cancer risk persisted (SIR = 1.08, 95% CI, 1.01‐1.15).

### 3.1 Colorectal cancer

During the first year, 0.3% (95% CI, 0.2%‐0.3%) of patients with fecal incontinence received a colorectal cancer diagnosis. It increased to 0.9% (95% CI, 0.7%‐1.1%) after 10 years (Table 2). The risks increased with advancing age, with colorectal cancer diagnoses reaching 0.7% (95% CI, 0.5%‐0.9%) after 1 year of follow‐up for patients older than 65 years.

**Table 2:** Cumulative incidences (%) of any cancer and other types of cancers after a first‐time hospital‐based diagnosis of fecal incontinence

	1 y, % (95% CI)	5 y, % (95% CI)	10 y, % (95% CI)
Any cancer^a^	1.4 (1.2‐1.6)	5.9 (5.5‐6.3)	10.8 (10.2‐11.5)
Age group (y)			
0‐17	0.1 (0.0‐0.2)	0.2 (0.1‐0.4)	0.3 (0.1‐0.6)
18‐34	0.1 (0.0‐0.4)	0.8 (0.4‐1.3)	2.3 (1.5‐3.4)
35‐49	0.6 (0.3‐1.0)	2.7 (2.0‐3.5)	5.3 (4.1‐6.6)
50‐64	1.7 (1.3‐2.2)	7.5 (6.5‐8.6)	15.2 (13.5‐17.0)
65+	3.1 (2.6‐3.6)	12.0 (11.0‐13.0)	20.8 (19.4‐22.3)
Sex			
Female	1.5 (1.2‐1.7)	6.1 (5.6‐6.6)	12.0 (11.2‐12.8)
Male	1.3 (1.0‐1.7)	5.4 (4.7‐6.1)	8.4 (7.4‐9.4)
Colorectal cancer	0.3 (0.2‐0.3)	0.6 (0.5‐0.8)	0.9 (0.7‐1.1)
Age group (y)			
0‐17	(.‐.)	(.‐.)	(.‐.)
18‐34	(.‐.)	(.‐.)	(.‐.)
35‐49	(.‐.)	0.1 (0.0‐0.4)	0.1 (0.0‐0.4)
50‐64	0.2 (0.1‐0.4)	0.6 (0.4‐1.0)	0.8 (0.5‐1.2)
65+	0.7 (0.5‐0.9)	1.5 (1.2‐1.9)	2.3 (1.8‐2.8)
Sex			
Female	0.2 (0.1‐0.3)	0.6 (0.4‐0.7)	0.9 (0.7‐1.2)
Male	0.4 (0.2‐0.6)	0.7 (0.5‐1.0)	0.9 (0.6‐1.2)
Other gastrointestinal cancer	0.1 (0.1‐0.2)	0.3 (0.2‐0.4)	0.6 (0.5‐0.8)
Age group (y)			
0‐17	(.‐.)	(.‐.)	(.‐.)
18‐34	(.‐.)	(.‐.)	(.‐.)
35‐49	0.0 (0.0‐0.3)	0.2 (0.1‐0.5)	0.2 (0.1‐0.5)
50‐64	0.2 (0.1‐0.4)	0.4 (0.2‐0.8)	1.0 (0.6‐1.6)
65+	0.2 (0.1‐0.4)	0.7 (0.5‐0.9)	1.3 (0.9‐1.7)
Sex			
Female	0.1 (0.1‐0.2)	0.3 (0.2‐0.4)	0.7 (0.5‐0.9)
Male	0.1 (0.0‐0.2)	0.4 (0.2‐0.6)	0.5 (0.3‐0.8)
Hormone‐related cancers	0.3 (0.2‐0.4)	1.3 (1.1‐1.5)	2.3 (2.0‐2.6)
Age group (y)			
0‐17	(.‐.)	(.‐.)	(.‐.)
18‐34	(.‐.)	0.1 (0.0‐0.4)	0.6 (0.2‐1.3)
35‐49	0.2 (0.1‐0.5)	0.7 (0.4‐1.2)	1.4 (0.8‐2.1)
50‐64	0.3 (0.2‐0.6)	2.0 (1.5‐2.5)	3.8 (2.9‐4.7)
65+	0.6 (0.4‐0.8)	2.3 (1.9‐2.8)	4.0 (3.4‐4.8)
Sex			
Female	0.3 (0.2‐0.4)	1.4 (1.2‐1.7)	2.7 (2.3‐3.2)
Male	0.3 (0.1‐0.4)	0.9 (0.6‐1.2)	1.4 (1.0‐1.9)
Lymphoma	0.1 (0.0‐0.1)	0.2 (0.2‐0.3)	0.4 (0.3‐0.6)
Age group (y)			
0‐17	(.‐.)	0.0 (0.0‐0.2)	0.1 (0.0‐0.3)
18‐34	(.‐.)	0.1 (0.0‐0.5)	0.1 (0.0‐0.5)
35‐49	(.‐.)	0.1 (0.0‐0.3)	0.2 (0.0‐0.6)
50‐64	0.1 (0.0‐0.3)	0.2 (0.1‐0.5)	0.4 (0.2‐0.8)
65+	0.2 (0.1‐0.3)	0.5 (0.3‐0.7)	0.8 (0.5‐1.2)
Sex			
Female	0.1 (0.0‐0.1)	0.2 (0.1‐0.3)	0.4 (0.3‐0.6)
Male	0.1 (0.0‐0.2)	0.3 (0.1‐0.5)	0.3 (0.2‐0.6)

CI: confidence interval.

(.‐.) Insufficient for estimates.

a The numbers do not sum up to the overall estimate for any cancer, because overall 10‐year risks of lung cancer (1%), basal cell carcinoma (2%), and a range of uncommon cancers were not included in any of the main cancer groups (as their cumulative incidences were low).

The SIR for colorectal cancer during the whole follow‐up period was 1.03 (95% CI, 0.84‐1.24). The 1‐year SIR for colorectal cancer was 2.31 (95% CI, 1.65‐3.13), while the SIR beyond 1 year was 0.77 (95% CI, 0.59‐0.98). The 1‐year SIR was higher for rectal cancer (3.67, 95% CI, 2.24‐5.67) than it was for colon and rectosigmoid cancer (1.70, 95% CI, 1.05‐2.60). The opposite pattern was observed for SIRs beyond 1 year (SIR for rectal cancer, 0.50, 95% CI, 0.27‐0.86 and SIR for colon and rectosigmoid cancer, 0.88, 95% CI, 0.66‐1.15).

### 3.2 Other gastrointestinal cancers

In total, 16 patients received a diagnosis of another gastrointestinal cancer within the year following their fecal incontinence diagnosis (Table 2). The 1‐year cumulative incidence was 0.1% (95% CI, 0.1%‐0.2%) and the 10‐year cumulative incidence was 0.6% (95% CI, 0.5%‐0.8%). The SIR for other gastrointestinal cancers during the whole follow‐up period was 1.13 (95% CI, 0.88‐1.44) and the 1‐year SIR was 1.56 (95% CI, 0.89‐2.54). No association was found beyond 1 year of follow‐up (SIR, 1.04, 95% CI, 0.77‐1.37).

### 3.3 Hormone‐related cancers and lymphoma

During the first year of follow‐up, 42 patients were diagnosed with a hormone‐related cancer, corresponding to a 1‐year cumulative incidence of 0.3% (95% CI, 0.2%‐0.4%). The SIR during the whole follow‐up period was 0.97 (95% CI, 0.85‐1.11), while the 1‐year SIR was 1.00 (95% CI, 0.72‐1.35). In the first year, 11 patients were diagnosed with lymphoma, equivalent to a 1‐year cumulative incidence of 0.1% (95% CI, 0.0%‐0.1%). The overall SIR of lymphoma was 1.35, 95% CI, 0.97‐1.81 and the 1‐year SIR was 2.02, 95% CI, 1.01‐3.61 (Table 3). Beyond 1 year of follow‐up, the SIR for hormone‐related cancers was unchanged (0.97, 95% CI, 0.84‐1.11). The SIR for lymphoma was 1.21, 95% CI, 0.83‐1.71).

**Table 3:** Observed and expected cancers and standardized incidence ratios (SIRs) for selected cancers after a first‐time hospital‐based diagnosis of fecal incontinence

	<1 y			≥1 y			All years		
	Observed cancers	Expected cancers	SIR (95% CI)	Observed cancers	Expected cancers	SIR (95% CI)	Observed cancers	Expected cancers	SIR (95% CI)
All cancers	225	170	1.32 (1.16‐1.51)	893	826	1.08 (1.01‐1.15)	1118	997	1.12 (1.06‐1.19)
Colorectal cancer	41	18	2.31 (1.65‐3.13)	66	86	0.77 (0.59‐0.98)	107	104	1.03 (0.84‐1.24)
Colon incl. rectosigmoid cancer	21	12	1.70 (1.05‐2.60)	53	60	0.88 (0.66‐1.15)	74	73	1.02 (0.80‐1.28)
Rectum	20	5	3.67 (2.24‐5.67)	13	26	0.50 (0.27‐0.86)	33	31	1.06 (0.73‐1.48)
Other gastrointestinal cancer	16	10	1.56 (0.89‐2.54)	51	49	1.04 (0.77‐1.37)	67	59	1.13 (0.88‐1.44)
Stomach	3	2	1.48 (0.31‐4.33)	11	9	1.19 (0.59‐2.12)	14	11	1.24 (0.68‐2.08)
Pancreas	5	4	1.30 (0.42‐3.03)	23	19	1.21 (0.77‐1.82)	28	23	1.23 (0.82‐1.78)
Hormone‐related cancers	42	42	1.00 (0.72‐1.35)	194	200	0.97 (0.84‐1.11)	236	242	0.97 (0.85‐1.11)
Breast	24	25	0.97 (0.62‐1.45)	123	122	1.01 (0.84‐1.21)	147	146	1.00 (0.85‐1.18)
Corpus uteri	2	5	0.44 (0.05‐1.60)	24	22	1.09 (0.70‐1.63)	26	26	0.98 (0.64‐1.44)
Ovary	4	3	1.24 (0.34‐3.19)	12	15	0.79 (0.41‐1.37)	16	18	0.87 (0.49‐1.41)
Prostate	12	9	1.28 (0.66‐2.24)	35	41	0.86 (0.60‐1.20)	47	50	0.94 (0.69‐1.25)
Lymphoma	11	5	2.02 (1.00‐3.61)	32	26	1.21 (0.83‐1.71)	43	32	1.35 (0.97‐1.81)
Non‐Hodgkin lymphoma	11	5	2.14 (1.07‐3.82)	29	25	1.16 (0.78‐1.67)	40	30	1.33 (0.95‐1.81)
Other cancers									
Larynx	2	0.7	2.84 (0.34‐10.25)	8	3	2.59 (1.12‐5.11)	10	4	2.64 (1.26‐4.85)
Lung	22	17	1.29 (0.81‐1.95)	92	80	1.14 (0.92‐1.40)	114	98	1.17 (0.96‐1.40)
Malignant melanoma	9	5	1.87 (0.86‐3.56)	28	24	1.18 (0.78‐1.70)	37	29	1.29 (0.91‐1.78)
Kidney	3	2	1.39 (0.29‐4.05)	18	10	1.78 (1.05‐2.81)	21	12	1.71 (1.06‐2.61)
Urinary bladder	7	6	1.13 (0.45‐2.32)	31	28	1.11 (0.76‐1.58)	38	34	1.12 (0.79‐1.53)
Membrane of the brain and spinal meninges	6	1	4.10 (1.50‐8.93)	8	7	1.08 (0.46‐2.12)	14	9	1.58 (0.86‐2.64)
Brain	2	3	0.78 (0.09‐2.83)	12	12	0.99 (0.51‐1.73)	14	15	0.95 (0.52‐1.60)
Metastases and nonspecified cancer in lymph nodes	6	3	1.84 (0.68‐4.01)	30	15	1.94 (1.31‐2.77)	36	19	1.92 (1.35‐2.66)

Abbreviations: CI, confidence interval; SIR, standardized incidence ratio.

Data on cancers less than 10 cases are not presented in the table.

### 3.4 Other cancers

Few patients were diagnosed with cancers other than those discussed above. Therefore, the associated SIR estimates were relatively imprecise. This was particularly true during the first year of follow‐up. Beyond 1 year, fecal incontinence was associated with increased risk of larynx cancer (SIR, 2.59, 95% CI, 1.12‐5.11) and kidney cancer (SIR, 1.78, 95% CI, 1.05‐2.81). A weak association with lung cancer also was observed (SIR, 1.14, 95% CI, 0.92‐1.40).

### 3.5 Subgroup analyses

We conducted subgroup analysis of patients who did/did not undergo colonoscopy during the 3‐month period prior to and 4 weeks after their fecal incontinence diagnosis, but due to few events the estimates were imprecise. The SIR for colorectal cancer during the first year of follow‐up was 0.98 (95% CI, 0.12‐3.54) in patients who underwent colonoscopy. However, it was slightly elevated in patients who did not have this procedure (1.19, 95% CI, 0.69‐1.90, Table 4). Beyond 1 year of follow‐up, the corresponding estimates were 0.24 (95% CI, 0.03‐0.86) among patients who underwent the procedure and 0.82 (95% CI, 0.63‐1.05) in patients who did not (Table 4).

**Table 4:** Observed and expected cancers and standardized incidence rates (SIRs) for colorectal cancers in patients with a first‐time hospital‐based diagnosis of fecal incontinence, by presence/absence of endoscopy or surgery

	<1 y			≥1 y			All years		
	Observed cancers	Expected cancers	SIR (95% CI)	Observed cancers	Expected cancers	SIR (95% CI)	Observed cancers	Expected cancers	**SIR (95% CI)**
Colonoscopy									
No	17	14	1.19 (1.69‐1.90)	64	78	0.82 (0.63‐1.05)	81	92	0.88 (0.70‐1.09)
Yes	2	2	0.98 (0.12‐3.54)	2	8	0.24 (0.03‐0.86)	4	10	0.38 (0.10‐0.98)
Sigmoidoscopy									
No	15	13	1.18 (0.03‐1.94)	66	71	0.93 (0.72‐1.19)	81	83	0.97 (0.77‐1.21)
Yes	4	4	1.12 (0.30‐2.86)	0	15	(.‐.)	4	19	0.21 (0.06‐0.54)
Lower surgery^a^									
No	17	15	1.10 (0.64‐1.76)	63	82	0.77 (0.59‐0.99)	80	97	0.82 (0.65‐1.02)
Yes	2	1	2.37 (0.29‐8.57)	3	4	0.70 (0.14‐2.04)	5	5	0.97 (0.32‐2.27)

Abbreviations: CI, confidence interval; SIR, standardized incidence ratio.

(.‐.) Insufficient for estimates.

a Lower surgery encompassed anal/perianal surgery, colectomy, surgery including the prostate, and surgery to repair rupture of the perineum.

After stratifying by sex, age at fecal incontinence diagnosis, type of hospital contact, and calendar period, the SIR estimates for colorectal cancer and other GI cancers remained fairly consistent with the main results (Table S5). Most associations were present in both patients with primary and secondary fecal incontinence diagnoses. Although the CIs were relatively wide, the associations were slightly stronger in patients with secondary diagnoses than in patients with primary diagnoses. The risk of colorectal cancer persisted after 1 year of follow‐up only for patients with high Charlson Comorbidity Index scores (SIR, 2.19, 95% CI, 1.17‐3.75).

## 4 DISCUSSION

In our population‐based cohort of 16,556 patients, fecal incontinence was a marker for cancer during nearly 20 years of follow‐up. This was mainly driven by an increased risk of colorectal cancer, other gastrointestinal cancers, and lymphoma diagnosed within the first year following a fecal incontinence diagnosis, which presumably is driven partly by reverse causation. However, the absolute cancer risks were low. The associations with lymphoma and other gastrointestinal cancers tapered off over time, while the risk of colorectal cancer was reduced beyond 1 year.

Gastrointestinal cancers are a major public health issue, representing about 18% of all incident cancers in the United States in 2018.^17^ Hence, identifying clinical markers of gastrointestinal cancers is of great interest. Lower gastrointestinal bleeding^6^ and constipation^18^ are well‐known presenting symptoms of colorectal cancers. In addition, a previous study found that unexplained chest pain/epigastric pain was associated with increased risk of gastrointestinal cancers.^19^ Our current study adds to the literature by quantifying the cancer risk associated with fecal incontinence. In a community‐dwelling cohort of 7250 patients with fecal incontinence adults aged 65 years or older in New Zealand, fecal incontinence was associated with increased mortality (hazard ratio = 1.26; no CIs were reported), after adjustment for age, sex, socioeconomic status, prior comorbidity, and prior cancer.^20^ The study did not report any nonfatal outcome data on cancer occurrence. We speculate that gastrointestinal infections and occurrence of some cancers after the index date may explain part of the increased mortality rate observed in these patients.

In our study, in addition to being a marker of colorectal cancer in the short term, fecal incontinence was associated with increased risk of a diagnosis of other gastrointestinal cancers, lymphoma, and kidney cancer during 1 year of follow‐up. These cancers likely represent prevalent but undiagnosed cancers that may cause fecal incontinence through local invasion or metastases to the perineum or that may lead to spinal cord compression. For example, our findings of increased colorectal cancers during the first year after the fecal incontinence likely represent reverse causation; that is, colorectal cancer causing fecal incontinence before the cancer becomes clinically overt. In support of this notion, the finding of a high rate of colonoscopy and sigmoidoscopy indicates that patients presented with other symptoms indicative of cancer, that is, pain, weight loss, or blood in the stool.

Although the CIs of long‐term SIR estimates were relatively wide, fecal incontinence also was a marker of incident cancers diagnosed after 1 year of follow‐up, including kidney cancer, lung cancer, and larynx cancer, which may be explained by shared risk factors such as smoking and obesity. For example, obesity is a risk factor for both fecal incontinence and kidney cancer.^8^ Interestingly, we found that colorectal cancer risk persisted over the long‐term among patients with high levels of comorbidity, suggesting that comorbidity may interact with fecal incontinence. Accordingly, patients with fecal incontinence and high levels of comorbidity also may be at high risk for cancer over the long term.

Frequent use of endoscopy or other diagnostic procedures can reveal gastrointestinal cancers or lead to a comprehensive diagnostic work‐up that identifies cancers. Our results for the first year of follow‐up therefore likely are influenced partly by heightened diagnostic efforts, as the initial increased risk of cancer during the first year was succeeded by a compensatory deficit for colorectal cancers, particularly for rectal cancers. We did not see such a pattern for the other cancers. However, as we also found a slightly increased SIR for colorectal cancer among patients who did not undergo endoscopy, our results are presumably not explained fully by intensified diagnostic efforts.

Clinically, it is important to evaluate whether patients presenting with fecal incontinence should receive a diagnostic work‐up for cancer. According to the most recent Clinical Practice Guideline for the Treatment of Fecal Incontinence from the American Society of Colon and Rectal Surgeons,^21^ endoscopy should be performed only in patients who have other specific symptoms of concern, for example, bleeding, urgency, tenesmus, and mucus drainage that may contribute to fecal incontinence and are indicative of colorectal cancer (grade of recommendation, 1B based on moderate‐quality evidence). In support of this recommendation and based on the relatively low absolute risks of cancers observed in our study, we do not suggest that routine endoscopy would lead to substantially increased or earlier detection of cancers in the absence of cancer‐related symptoms and signs. The number of endoscopies needed to detect additional cancers would likely be high and not cost‐effective. Opportunistic screening should be considered only if specific accompanying symptoms indicative of a cancer are present.

### 4.1 Strengths and limitations

The strengths of this study include its nationwide population‐based design based on the uniform Danish health care system, as well as its long‐term follow‐up of patients. Danish administrative and health registries have high completeness and virtually no loss to follow‐up, minimizing the risk of selection bias. Cancer diagnoses have high accuracy and completeness in the DCR, with 89% of tumors verified morphologically.^12^ No previous study has validated the fecal incontinence diagnosis in the DNPR. However, we expect a high positive predictive value, as an inpatient or outpatient hospital contact would require a preceding visit to a primary care physician with a hospital referral. It is important to note that the patients with fecal incontinence included in our study presumably represent the most severe cases, which required hospitalization or referral to a hospital outpatient clinic. Thus our results may not necessarily translate to all patients with fecal incontinence. In addition, as the exact date of first fecal incontinence symptoms is not captured by the registries and as many patients had a fecal incontinence diagnosis during a hospital stay for another reason, the analyses stratified by follow‐up period should be interpreted with caution. Fecal incontinence has major social impact and severely affects quality of life. Therefore, it is likely that fecal incontinence leads to medical contact close to first onset, and that we follow our cohort from recent exposure.

## 5 CONCLUSIONS

In this population‐based study, fecal incontinence was a marker of cancer during nearly 20 years of follow‐up. The incidence of gastrointestinal cancer and lymphoma diagnoses during 1 year of follow‐up was higher among patients with fecal incontinence than expected in the general population, which could be partly explained by reverse causation. However, the absolute risks were low.

## CONFLICT OF INTEREST

None.

## Data Availability

The data, analytic methods, and other study materials will not be made available to other researchers for purposes of reproducing the results or replicating the study. Such disclosure would conflict with regulations for use of Danish health care data.
