# Supplementary material for: Risk of cancer in patients with fecal incontinence
Source: Cancer Med. 2019 Aug 29;8(14):6449–57. doi: 10.1002/cam4.2509 (PMC6797632; doi:10.1002/cam4.2509)
Supplement: Supplementary file 1 [file CAM4-8-6449-s001.docx]

**Supplementary Table 1.** ICD codes used in the study.

|  | ICD-10 |
| --- | --- |
| **Fecal incontinence** | R15, 78569 (ICD-8) |
| **Cancers** |  |
| **Any cancer** | All C-codes |
| **Colorectal cancer** | C18-20 |
| Colon incl. rectosigmoid cancer | C18-C19 |
| Rectum | C20 |
| **Other gastrointestinal cancer** | C15-17, C21-26 |
| Esophagus | C15 |
| Stomach | C16 |
| Small intestine | C17 |
| Anal | C21, excl. morphology codes 809, 872-879 |
| Liver | C22 |
| Gallbladder and biliary tracts | C23-C24 |
| Pancreas | C25 |
| **Hormone-related cancer** | C50, C54-56, C61-62, C73 |
| Breast | C50 |
| Corpus uteri | C54–C55 |
| Ovary | C56 |
| Prostate | C61 |
| **Lymphoma** | C81-86, C90 |
| Hodgkin | C81, morphology codes 965-966 |
| Non-Hodgkin | C82–C85, C90, morphology 959, 967-972 |
| **Other cancers** |  |
| Oral cavity | C03–06 |
| Larynx | C32 |
| Lung | C33-C34 |
| Malignant melanoma | C43, C21 if morphology codes 879-879 |
| Kidney | C64 |
| Urinary bladder | C67, and D090, D303, D414 if morphology codes 812-813 |
| Membrane of the brain and spinal meninges | C70 ,D32,D42 |
| Brain | C71, C751–C753, D330–D332, D352–D354, D430–D432, D443–D445 |
| Metastases and non-specified cancer in lymph nodes | C77–C79 |

Abbreviation: ICD, *International Classification of Diseases*

| Supplementary Table 2. Codes for conditions included in the modified Charlson Comorbidity Index. | | | |
| --- | --- | --- | --- |
| Comorbidities | **Weight** | **ICD-8** | ICD-10 |
| Myocardial infarction | 1 | 410 | I21; I22; I23 |
| Congestive heart failure |  | 427.09; 427.10; 427.11; 427.19; 428.99; 782.49 | I50; I11.0; I13.0; I13.2 |
| Peripheral vascular disease |  | 440; 441; 442; 443; 444; 445 | I70; I71; I72; I73; I74; I77 |
| Cerebrovascular disease |  | 430–438 | I60-I69; G45; G46 |
| Dementia |  | 290.09-290.19; 293.09 | F00-F03; F05.1; G30 |
| Chronic pulmonary disease |  | 490–493; 515-518 | J40-J47; J60-J67; J68.4; J70.1;  J70.3; J84.1; J92.0; J96.1; J98.2; J98.3 |
| Connective tissue disease |  | 712; 716; 734; 446; 135.99 | M05; M06; M08; M09; M30; M31;  M32; M33; M34; M35; M36; D86 |
| Ulcer disease |  | 530.91; 530.98; 531–534 | K22.1; K25–K28 |
| Mild liver disease |  | 571; 573.01; 573.04 | B18; K70.0–K70.3; K70.9; K71; K73; K74; K76.0 |
| Diabetes without end-organ damage |  | 249.00, 249.06, 249.07, 249.09, 250.00, 250.06, 250.07, 250.09 | E10.0, E10.1, E10.9, E11.0, E11.1, E11.9 |
| Diabetes with end-organ damage | 2 | 249.01-249.05, 249.08, 250.01-250.05, 250.08 | E10.2- E10.8, E11.2-E11.8 |
| Hemiplegia |  | 344 | G81; G82 |
| Moderate to severe renal disease |  | 403; 404; 580–583; 584; 590.09; 593.19; 753.10–753.19; 792 | I12; I13; N00–N05; N07; N11; N14; N17–N19; Q61 |
| Moderate to severe liver disease | 3 | 070.00; 070.02; 070.04; 070.06; 070.08; 573.00; 456.00-456.09 | B15.0; B16.0; B16.2; B19.0; K70.4; K72; K76.6; I85 |
| Acquired immune deficiency syndrome | 6 | 079.83 | B21–B24 |

Abbreviations: ICD, International Classification of Diseases.

**Supplementary Table 3**. Procedure and surgery codes used in the study.

|  | **1977-1995** | **1996-2013** |
| --- | --- | --- |
| Colonosopy and sigmoidoscopy | 91070, 91080 | KUJF32- KUJF35  KUJF42–45, KJFA15 – KJGA05 |
| Lower surgery* | 43785, 43786, 43890, 45130, 45405, 45700-46061, 46100, 46120-46232, 46280-46291, 46320-46340, 46360, 46400, 46460-46470, 46500-46520, 46540-46990, 49300-49610  55020 (19730401-19881231)  55040-55080,  55100 (19730401-19801231)  55120,  55150 (19810101-19881231)  55151, 55160-55180,  55200 (19730401-19881231)  55200 (19890101-19951231)  55220, 55239  66440-66460, 66110, 45060-45081, 46530 | KJFG53-56, KJFG70-96,  KJG  KJH  KJNA  KLFC96, KLFE99  KKCC10-20, KKEC, KKED, KMBC  KJFH |
| Episiotomy | 65400 | KTMD |

*Lower surgery encompassed anal/perianal surgery, colectomy, surgery including the prostate, and surgery to repair rupture of the perineum.

| **Supplementary Table 4.** ICD-codes used in the study. | | |
| --- | --- | --- |
|  | **ICD-8** | **ICD-10** |
| Inflammatory bowel disease | 56319,56904, 56301 | K50, K510-K513 |
| Parkinson’s disease | 342 | G20 |
| Diabetes mellitus | 249, 250 (excluding 249.02, 250.02) | E10 (excluding E10.2), E11 (excluding E11.2), H36.0 |
| Obesity | 277 | E65-E68 |
| Child birth | 650-666 | O80-84 |
| Abbreviations: ICD, *International Classification of Diseases*; ATC, Anatomical Therapeutic Chemical classification system. | | |

| **Supplementary Table 5.** Standardized incidence rates (SIRs) with 95% confidence intervals (CIs) for cancers among patients with a first-time diagnosis of fecal incontinence, by age, sex, type of hospital contact, calendar period, Charlson Comorbidity Index score, and child birth. | | | | | |
| --- | --- | --- | --- | --- | --- |
|  | **All cancers** | **Colorectal cancer** | **Other gastrointestinal cancer** | **Hormone**  **related cancers** | **Lymphoma** |
| **<1 year from diagnosis** | | |  |  |  |
| **Age groups** |  |  |  |  |  |
| 0-17 | 3.91 (0.47-14.10) | (.-.) | (.-.) | (.-.) | (.-.) |
| 18-34 | 0.94 (0.11-3.38) | (.-.) | (.-.) | (.-.) | (.-.) |
| 35-49 | 1.49 (0.77-2.60) | (.-.) | 4.46 (0.11-24.82) | 1.64 (0.45-4.19) | (.-.) |
| 50-64 | 1.43 (1.07-1.87) | 2.49 (1.00-5.14) | 2.65 (0.86-6.16) | 0.87 (0.42-1.61) | 2.67 (0.55-7.80) |
| 65+ | 1.28 (1.08-1.49) | 2.32 (1.61-3.25) | 1.24 (0.59-2.27) | 1.01 (0.67-1.46) | 2.03 (0.88-4.00) |
| **Sex** |  |  |  |  |  |
| Female | 1.28 (1.09-1.50) | 1.68 (1.04-2.56) | 1.75 (0.90-3.06) | 0.90 (0.60-1.29) | 1.59 (0.58-3.46) |
| Male | 1.44 (1.12-1.82) | 3.80 (2.32-5.86) | 1.19 (0.32-3.03) | 1.35 (0.72-2.32) | 3.01 (0.97-7.01) |
| **Type of hospital contact** |  |  |  |  |  |
| Inpatient | 1.95 (1.38-2.68) | 4.48 (2.14-8.23) | 4.70 (1.72-10.24) | 0.69 (0.14-2.00) | 1.60 (0.04-8.91) |
| Outpatient | 1.24 (1.07-1.43) | 1.99 (1.35-2.83) | 1.12 (0.53-2.05) | 1.04 (0.74-1.42) | 2.07 (0.99-3.81) |
| Primary diagnoses | 1.23 (1.05-1.43) | 2.19 (1.50-3.10) | 1.31 (0.65-2.34) | 0.88 (0.60-1.26) | 1.99 (0.91-3.79) |
| Secondary diagnoses | 1.78 (1.33-2.33) | 2.81 (1.29-5.34) | 2.76 (0.90-6.44) | 1.60 (0.80-2.86) | 2.15 (0.26-7.77) |
| **Calendar period** |  |  |  |  |  |
| 1995–2001 | 1.37 (0.98-1.86) | 1.96 (0.78-4.03) | 0.96 (0.12-3.47) | 1.34 (0.64-2.46) | 2.98 (0.61-8.70) |
| 2002–2009 | 1.38 (1.15-1.65) | 2.33 (1.44-3.56) | 1.97 (0.94-3.62) | 0.87 (0.52-1.36) | 1.82 (0.59-4.24) |
| 2010–2013 | 1.20 (0.92-1.53) | 2.50 (1.33-4.27) | 1.30 (0.35-3.34) | 1.03 (0.55-1.76) | 1.77 (0.37-5.18) |
| **CCI score** |  |  |  |  |  |
| Low (0) | 1.21 (1.00-1.46) | 1.85 (1.08-2.96) | 1.68 (0.77-3.20) | 0.83 (0.51-1.29) | 1.67 (0.54-3.89) |
| Moderate (1-2) | 1.40 (1.11-1.73) | 2.75 (1.63-4.35) | 1.34 (0.43-3.13) | 1.37 (0.83-2.14) | 2.67 (0.86-6.22) |
| High (3+) | 1.67 (1.13-2.39) | 2.93 (1.07-6.38) | 1.72 (0.21-6.22) | 0.74 (0.15-2.17) | 1.73 (0.04-9.64) |
| **Child birth** |  |  |  |  |  |
| No | 1.33 (1.16-1.51) | 2.31 (1.66-3.14) | 1.57 (0.90-2.55) | 1.00 (0.71-1.35) | 2.06 (1.03-3.69) |
| Yes | 1.23 (0.33-3.14) | (.-.) | (.-.) | (.-.) | (.-.) |
| **≥1 year from diagnosis** | | |  |  |  |
| **Age groups** |  |  |  |  |  |
| 0-17 | 1.90 (0.70-4.13) | (.-.) | (.-.) | (.-.) | 3.71 (0.45-13.39) |
| 18-34 | 1.13 (0.71-1.72) | (.-.) | (.-.) | 1.13 (0.36-2.62) | 3.43 (0.42-12.40) |
| 35-49 | 1.02 (0.78-1.31) | 0.69 (0.08-2.49) | 0.96 (0.12-3.45) | 0.85 (0.49-1.38) | 1.26 (0.15-4.54) |
| 50-64 | 1.06 (0.94-1.21) | 0.62 (0.32-1.09) | 1.11 (0.61-1.86) | 0.93 (0.72-1.19) | 0.85 (0.31-1.85) |
| 65+ | 1.09 (1.00-1.18) | 0.82 (0.61-1.07) | 1.03 (0.72-1.43) | 1.01 (0.83-1.22) | 1.20 (0.73-1.85) |
| **Sex** |  |  |  |  |  |
| Female | 1.08 (1.00-1.17) | 0.78 (0.58-1.03) | 1.01 (0.70-1.40) | 1.00 (0.85-1.17) | 1.25 (0.80-1.86) |
| Male | 1.07 (0.94-1.22) | 0.73 (0.42-1.18) | 1.12 (0.64-1.82) | 0.84 (0.58-1.17) | 1.10 (0.47-2.17) |
| **Type of hospital contact** |  |  |  |  |  |
| Inpatient | 0.96 (0.76-1.20) | 0.34 (0.07-0.98) | 1.40 (0.56-2.89) | 0.75 (0.41-1.26) | 1.48 (0.40-3.79) |
| Outpatient | 1.09 (1.02-1.17) | 0.82 (0.63-1.04) | 1.00 (0.73-1.34) | 0.99 (0.85-1.15) | 1.18 (0.78-1.70) |
| Primary diagnoses | 1.09 (1.01-1.17) | 0.80 (0.61-1.03) | 1.00 (0.72-1.35) | 0.98 (0.84-1.14) | 1.27 (0.85-1.83) |
| Secondary diagnoses | 1.03 (0.86-1.24) | 0.57 (0.23-1.17) | 1.31 (0.60-2.48) | 0.86 (0.54-1.29) | 0.81 (0.17-2.37) |
| **Calendar period** |  |  |  |  |  |
| 1995–2001 | 1.09 (0.96-1.22) | 0.93 (0.60-1.36) | 1.01 (0.58-1.64) | 1.10 (0.85-1.39) | 1.58 (0.84-2.69) |
| 2002–2009 | 1.06 (0.98-1.16) | 0.73 (0.52-1.01) | 1.05 (0.71-1.49) | 0.86 (0.70-1.03) | 0.98 (0.56-1.60) |
| 2010–2013 | 1.20 (0.95-1.51) | 0.32 (0.04-1.16) | 1.11 (0.30-2.84) | 1.37 (0.84-2.12) | 1.52 (0.31-4.43) |
| **CCI score** |  |  |  |  |  |
| Low (0) | 1.01 (0.93-1.10) | 0.75 (0.54-1.02) | 0.88 (0.58-1.29) | 1.02 (0.86-1.21) | 1.12 (0.67-1.74) |
| Moderate (1-2) | 1.17 (1.04-1.32) | 0.49 (0.26-0.83) | 0.99 (0.55-1.63) | 0.84 (0.62-1.12) | 1.54 (0.80-2.70) |
| High (3+) | 1.33 (1.04-1.68) | 2.19 (1.17-3.75) | 2.70 (1.24-5.13) | 0.96 (0.48-1.72) | 0.59 (0.02-3.31) |
| **Child birth** |  |  |  |  |  |
| No | 1.09 (1.02-1.16) | 0.77 (0.60-0.98) | 1.05 (0.78-1.38) | 0.97 (0.84-1.12) | 1.16 (0.78-1.66) |
| Yes | 0.92 (0.59-1.36) | (.-.) | (.-.) | 0.82 (0.30-1.78) | 2.96 (0.36-10.67) |
| **All years** |  |  |  |  |  |
| **Age groups** |  |  |  |  |  |
| 0-17 | 2.18 (0.94-4.29) | (.-.) | (.-.) | (.-.) | 3.31 (0.40-11.95) |
| 18-34 | 1.11 (0.71-1.66) | (.-.) | (.-.) | 1.04 (0.34-2.41) | 2.95 (0.36-10.63) |
| 35-49 | 1.08 (0.84-1.36) | 0.62 (0.08-2.26) | 1.30 (0.27-3.79) | 0.94 (0.57-1.45) | 1.10 (0.13-3.97) |
| 50-64 | 1.12 (0.99-1.25) | 0.86 (0.52-1.35) | 1.31 (0.79-2.05) | 0.92 (0.72-1.16) | 1.10 (0.50-2.09) |
| 65+ | 1.12 (1.04-1.21) | 1.10 (0.88-1.36) | 1.07 (0.78-1.43) | 1.01 (0.85-1.19) | 1.36 (0.90-1.96) |
| **Sex** |  |  |  |  |  |
| Female | 1.12 (1.04-1.19) | 0.93 (0.72-1.17) | 1.13 (0.83-1.50) | 0.98 (0.85-1.14) | 1.30 (0.88-1.86) |
| Male | 1.14 (1.01-1.28) | 1.32 (0.92-1.83) | 1.14 (0.69-1.75) | 0.93 (0.69-1.24) | 1.46 (0.77-2.49) |
| **Type of hospital contact** |  |  |  |  |  |
| Inpatient | 1.15 (0.95-1.38) | 1.17 (0.62-2.00) | 2.07 (1.10-3.55) | 0.74 (0.43-1.18) | 1.50 (0.49-3.50) |
| Outpatient | 1.12 (1.05-1.19) | 1.01 (0.82-1.24) | 1.02 (0.77-1.33) | 1.00 (0.87-1.14) | 1.33 (0.94-1.82) |
| Primary diagnoses | 1.11 (1.04-1.18) | 1.03 (0.83-1.26) | 1.05 (0.79-1.37) | 0.97 (0.84-1.11) | 1.39 (0.98-1.91) |
| Secondary diagnoses | 1.19 (1.01-1.38) | 1.03 (0.59-1.67) | 1.61 (0.88-2.70) | 1.01 (0.70-1.41) | 1.08 (0.35-2.52) |
| **Calendar period** |  |  |  |  |  |
| 1995–2001 | 1.12 (1.00-1.25) | 1.04 (0.72-1.46) | 1.00 (0.60-1.59) | 1.12 (0.89-1.40) | 1.73 (0.99-2.81) |
| 2002–2009 | 1.11 (1.03-1.20) | 0.97 (0.74-1.25) | 1.18 (0.85-1.60) | 0.86 (0.71-1.02) | 1.11 (0.68-1.69) |
| 2010–2013 | 1.20 (1.01-1.42) | 1.32 (0.74-2.17) | 1.20 (0.52-2.36) | 1.21 (0.84-1.70) | 1.64 (0.60-3.57) |
| **CCI score** |  |  |  |  |  |
| Low (0) | 1.04 (0.96-1.13) | 0.91 (0.69-1.18) | 1.00 (0.70-1.39) | 0.99 (0.84-1.16) | 1.20 (0.77-1.78) |
| Moderate (1-2) | 1.22 (1.10-1.35) | 0.93 (0.63-1.32) | 1.06 (0.65-1.63) | 0.94 (0.73-1.20) | 1.76 (1.03-2.82) |
| High (3+) | 1.42 (1.15-1.73) | 2.38 (1.43-3.72) | 2.45 (1.22-4.38) | 0.90 (0.49-1.52) | 0.88 (0.11-3.19) |
| **Child birth** |  |  |  |  |  |
| No | 1.13 (1.06-1.20) | 1.04 (0.85-1.25) | 1.14 (0.88-1.45) | 0.98 (0.85-1.11) | 1.32 (0.94-1.79) |
| Yes | 0.95 (0.64-1.37) | (.-.) | (.-.) | 0.87 (0.35-1.78) | 2.55 (0.31-9.22) |

Abbreviation: CCI, Charlson Comorbidity Index

(.-.) Insufficient for estimates
